# The bleeding heart: A case of cardiac angiosarcoma presenting with pulmonary hemorrhage and hemoptysis

**DOI:** 10.1016/j.radcr.2025.05.023

**Published:** 2025-06-09

**Authors:** Maher Marar, Saad Mohammad, Ahmad Abdulraheem, Munther Hammad, Sa’ed Al Hayek

**Affiliations:** a School of Medicine, The University of Jordan, Amman 11942, Jordan; b Department of Medicine, Medstar Washington Hospital Center, Washington, DC 20010, USA

**Keywords:** Angiosarcoma, Cardiac angiosarcoma, Pulmonary hemorrhage, Hemoptysis, Sarcoma, Heart

## Abstract

Primary cardiac angiosarcoma is an extremely rare and aggressive tumor that originates from the endothelial lining of cardiac blood vessels. We report a case of a 48-year-old man with history of military burn pit exposure, who presented with shortness of breath, cough, and hemoptysis. He was treated as a case of pneumonia, later he developed a diffuse pulmonary hemorrhage, and the etiology of his symptoms was found to be cardiac angiosarcoma with lung involvement. This case highlights the important clinical, radiological, and pathological characteristics of a rare cancer that commonly spreads to the lungs, urging healthcare providers to keep it in mind when diagnosing hemoptysis and pulmonary hemorrhage.

## Introduction

Angiosarcomas are rare tumors that originate from endothelial cells, and most commonly arise from the skin, breast, liver, or spleen [1]. Primary cardiac angiosarcoma is even rarer and an extremely aggressive subtype that originates from the endothelial lining of cardiac blood vessels [2].

The reported incidence of cardiac angiosarcoma in autopsies is 0.0001%-0.030% [1]. It has a very poor prognosis and the median survival of diagnosed patients is reported to be 13 months, with poorer prognosis in metastatic disease making a median overall survival of only 6 months [3].

We report a case of a 48-year-old man who presented with shortness of breath, cough, and hemoptysis and later developed pulmonary hemorrhage. He was initially treated as a case of pneumonia, but the etiology of his symptoms was later found to be cardiac angiosarcoma with lung involvement. Additionally, we discuss the differential diagnosis of this condition and highlight recent advances in therapeutic management.

## Case presentation

**Fig. 1:**
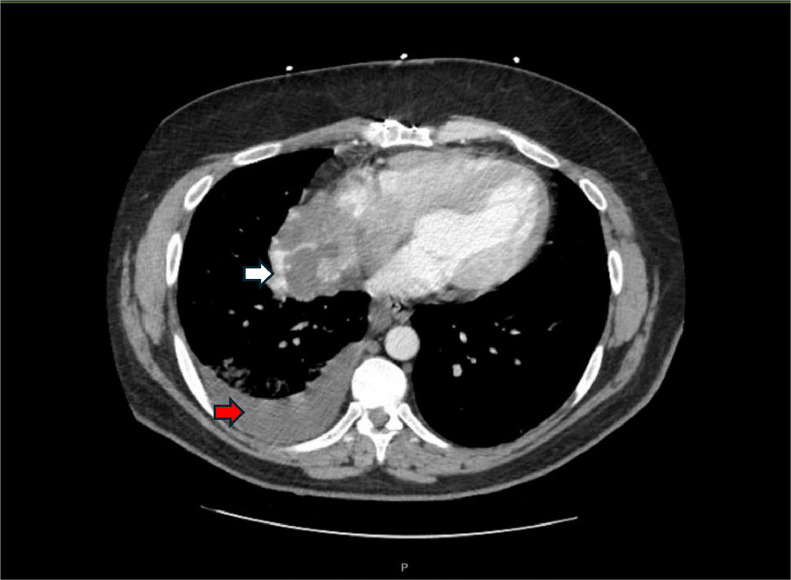
Axial contrast-enhanced CT chest showing a 6.6 × 6.3 cm irregular, heterogeneously enhancing, hypodense mass (white arrow) arising from the right atrial appendage. Moderate right pleural effusion is also noted (red arrow).

A 48-year-old white male with a history of bicuspid aortic valve replacement and Wolff-Parkinson-White syndrome (WPW) presented with a two-week history of progressive dyspnea, cough, and hemoptysis. Chest X-ray revealed consolidation, and despite multiple antibiotics and steroids, his symptoms worsened, prompting an emergency department visit. A contrast-enhanced axial CT chest was obtained and revealed a 6.6 × 6.3 cm irregular, hypodense, heterogeneously enhancing mass arising from the right atrial appendage, consistent with a hypervascular lesion. Associated findings included mediastinal lymphadenopathy, bilateral pulmonary nodules, and a moderate right pleural effusion (Fig. 1).

**Fig. 2:**
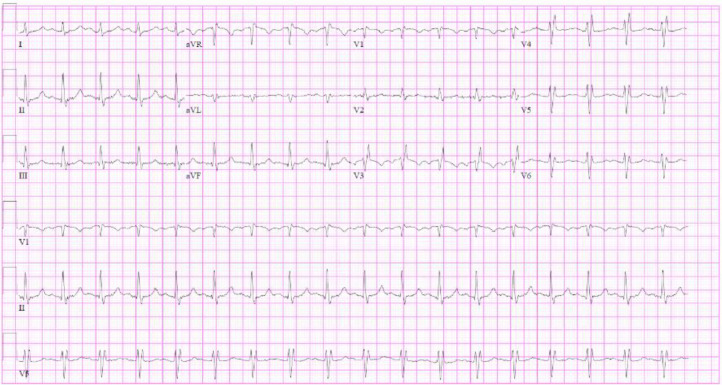
12-lead ECG on admission showing sinus tachycardia at 110 bpm with no ST-segment changes or arrhythmias.

The patient was subsequently admitted to the surgical intensive care unit (SICU) for worsening hypoxemia and for cardiac surgery evaluation. He was afebrile and denied any chest pain. His oxygen saturation was 94% on a 4L nasal cannula. Initial ECG on admission demonstrated sinus tachycardia at 110 bpm without ischemic changes (Fig. 2). Labs showed leukocytosis (WBC 12.74 k/µL), anemia (Hb 8.9 g/dL), and INR 5.0, which was corrected with vitamin K and PCC. His past history included exposure to military burn pits in Afghanistan, but he had no history of smoking, alcohol, or illicit drug use. Family history was notable only for a maternal grandmother with leukemia, with no familial cancer syndromes or autoimmune diseases.

**Fig. 3:**
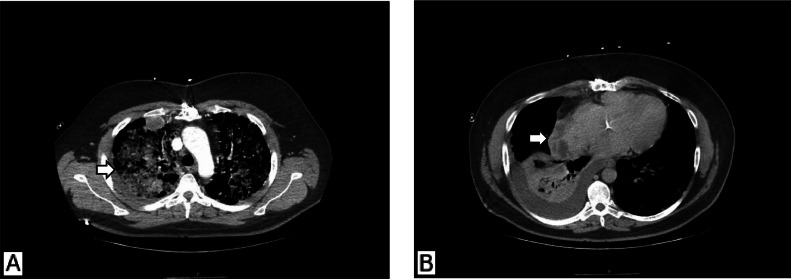
(A) Axial CT chest with contrast showing bilateral dense ground-glass opacities in upper and lower lobes, consistent with diffuse pulmonary hemorrhage (arrow). (B) The right atrial mass shows heterogeneous enhancement and is isoattenuating to the blood pool (arrow).

Three days after admission he developed night sweats and acute hypoxemic respiratory failure necessitating high-flow nasal cannula (HFNC) support. his hemoglobin dropped to 6.1 g/dL requiring RBC transfusion. repeat CT scan demonstrated scattered bilateral pleural and pulmonary metastatic nodules, diffuse bilateral dense ground-glass opacities concerning for pulmonary hemorrhage, and a right pleural effusion with basilar atelectasis (Fig. 3). A chest tube was placed draining 500cc of blood. A multidisciplinary team was consulted, and the differential diagnosis included primary cardiac sarcoma, lymphoma, metastatic bronchogenic carcinoma; infectious or inflammatory etiologies were less likely.

**Fig. 4:**
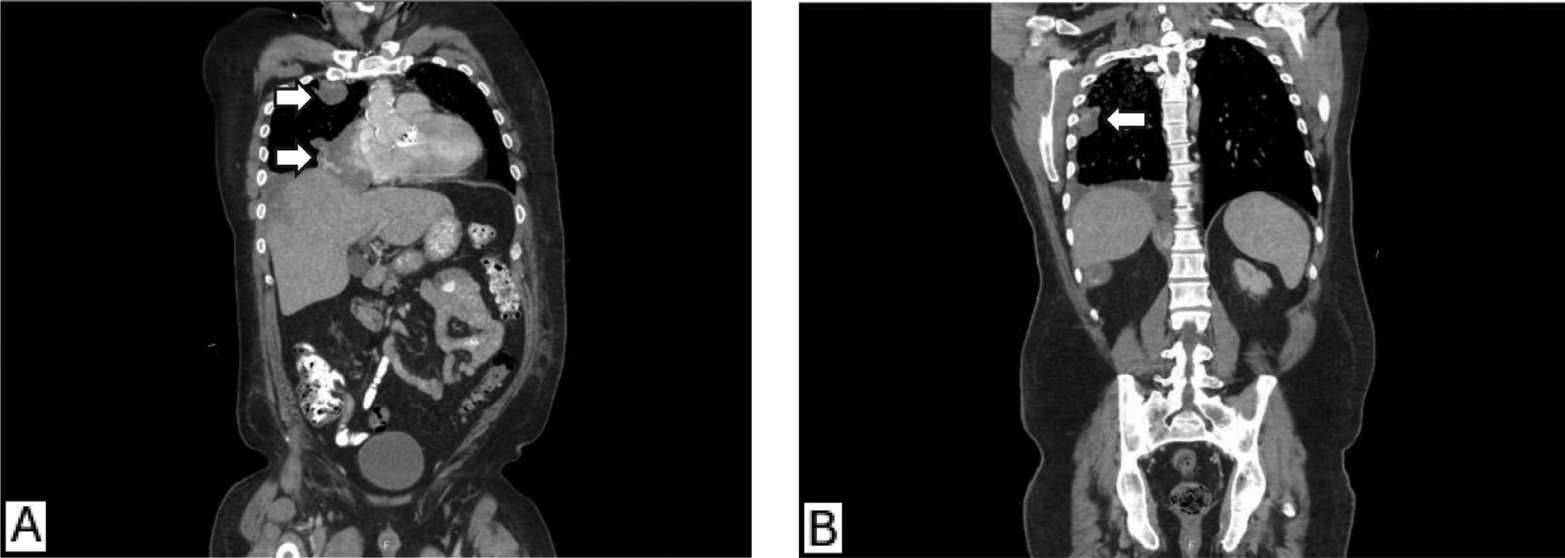
Coronal contrast-enhanced CT chest: (A) Large heterogeneously enhancing mass (7.6 × 6.8 cm) originating from the right atrial wall and extending along the right heart border. A 3.1 cm pleural metastasis is noted anterior to the right upper lobe. (B) Enhancing anterior right basilar pleural lesion (4.5 cm) with interval reduction in right pleural effusion and improvement in pulmonary ground-glass opacities.

Given the mass’s location, CT-guided biopsy was preferred over bronchoscopy but was delayed until the patient was stabilized. Pleural fluid analysis confirmed an exudative effusion, whereas the cultures and cytology results were negative. Flow cytometry and sputum culture did not reveal malignancy. Peripheral smear showed increased reticulocytes without hemolysis. A follow-up CT chest revealed a persistently enlarging right atrial mass (7.6 × 6.8 cm) and multiple pleural-based hypervascular metastases, a 3.1 cm right upper lobe pleural lesion, a 4.5 cm basilar pleural mass, and improvement in pulmonary ground-glass opacities (Fig. 4).

Five days later and after the patient's status stabilized, a CT-guided core biopsy of the right pleural mass was performed and showed epithelioid and spindle cell proliferation involving lung parenchyma, favoring angiosarcoma. Submitted immunohistochemical stains demonstrated that the lesional cells were positive for WT-1 (nuclear), CD34, beta-catenin (membranous), and ERG-2, and an increased Ki-67 proliferation index of 30%-40%.

Supportive management was provided, and he was advised to follow up with oncology. He was discharged per his request and remains stable off oxygen at the time of writing.

## Discussion

Primary cardiac angiosarcoma is a rare and extremely aggressive tumor that originates from the endothelial lining of cardiac blood vessels [2]. Most patients with cardiac angiosarcoma present with shortness of breath, pericardial effusion and chest pain. Additionally, metastasis is common at presentation and the most common locations are the lung, liver, and bones [4]. Our patient presented with a two-week history of shortness of breath and hemoptysis, and later developed pulmonary hemorrhage, which is an unusual presentation and has been reported in only a few cases in the literature [3,5].

Most cases of angiosarcoma are idiopathic. However, some risk factors described in the literature include previous exposure to radiation, chronic lymphoedema, environmental carcinogens and genetic syndromes [6]. Our patient had previous history of exposure to military burn pits in Afghanistan, which might have increased his risk of developing this condition.

The diagnosis of cardiac angiosarcoma involves a combination of imaging techniques and a tissue biopsy. Electrocardiography (ECG) often reveals nonspecific abnormalities that can mimic acute coronary syndromes [7]. Transesophageal echocardiography (TEE) is typically the initial diagnostic modality, as it has a sensitivity of 97% for identifying cardiac masses. However, owing to the high rates of metastasis at the time of diagnosis, it is essential to obtain a CT scan or cardiac MRI (superior to CT) as these modalities can provide a better understanding of the cardiac tumor anatomy and help detect systemic metastasis [2].

The differential diagnosis in our case included cardiac angiosarcoma, cardiac myxoma, primary cardiac lymphoma, and metastatic carcinoma. Table 1 summarizes these conditions, highlighting differences in clinical presentation and imaging characteristics.

**Table 1:** Imaging features and clinical characteristics of common cardiac masses.

Tumor type	Location	Imaging features in CT	Imaging features in MRI	Imaging features in ultrasound	Common clinical features	
Cardiac myxoma	Left atrium (interatrial septum near fossa ovalis)	Ovoid/lobular Heterogeneous enhancement Lower attenuation than myocardium	Heterogeneous enhancement post-gadolinium T2 hyperintensity	Mobile, pedunculated mass Attached to interatrial septum	Embolic events (e.g., stroke) Constitutional symptoms Mitral valve obstruction (dyspnea)	[8,9]
Cardiac angiosarcoma	Right atrium (broad-based, infiltrates pericardium, tricuspid valve, or coronary arteries)	Inhomogeneous density Heterogeneous centripetal enhancement Right coronary artery encasement common	"Cauliflower-like" morphology Heterogeneous T1/T2 signal (hemorrhage) Rim/patchy enhancement	Right atrial mass with invasive features	Right-sided heart failure Chest pain Pericardial effusion/tamponade Extremely poor prognosis	[10]
Primary cardiac lymphoma	Right heart chambers (often involving pericardium/great vessels)	hypo or isoattenuating soft-tissue mass Heterogeneous enhancement	T1 hypointense, T2 hyperintense Infiltrative growth pattern Pericardial involvement	Hypoechoic myocardial mass Pericardial effusion	Rapid progression Heart failure symptoms, Arrhythmias Poor prognosis without treatment	[11,12]
Metastatic carcinoma	Variable (depends on primary tumor; pericardium is the commonest site)	Variable (nodular/mass-like) Often multifocal	Depends on primary tumor Diffusion restriction may be seen	Variable (echogenic or hypoechoic) Often associated with effusions	Symptoms depend on metastatic site Often advanced primary malignancy Pericardial involvement	[13]

According to Kumaran et al., in all reported cases of cardiac angiosarcomas, pericardial fluid cytology has consistently yielded negative results [14]. Even in cases of Primary pleural Angiosarcoma pleural fluid cytology is usually negative [15]. Therefore, it is crucial to obtain a surgical biopsy with histopathological and immunohistochemical evaluation to confirm the diagnosis. Our patient had primary cardiac mass with lung parenchyma and pleural involvement, yet the pleural fluid cytology we obtained was negative for malignancy. The reason for the consistent negative cytology is unknown and was not investigated before in the literature.

Due to the rarity of this condition there is no strict guideline for its management. Complete surgical resection has the greatest impact on survival but is difficult to achieve given the visceral location of these tumors [16]. Tumors that are large, diffusely infiltrative or presenting with extensive metastases are typically considered inoperable, necessitating a shift toward systemic therapeutic strategies that are mostly palliative in nature [17]. A multimodal approach including surgery, chemotherapy, and radiation therapy has shown some benefit in increasing overall survival, with doxorubicin, paclitaxel, and ifosfamide being the most commonly used regimens [16,18].

Targeted therapies, notably VEGF inhibitors such as pazopanib, sorafenib, and imatinib, have demonstrated modest antitumor activity in angiosarcoma. Among these agents, pazopanib combines the most favorable efficacy, achieving a median progression-free survival of approximately 3 months and an overall survival near 9 months. Combination approaches, including taxanes, anti-VEGF agents, and radiotherapy, have yielded occasional long-term survival in case reports. Immunotherapy, particularly immune checkpoint inhibitors (e.g., anti-PD-1), is an emerging option with promising outcomes. Developing targeted therapies tailored to specific genetic alterations offers further potential to enhance treatment efficacy and improve patient outcomes in this aggressive malignancy [19].

## Conclusion

This case of primary cardiac angiosarcoma—initially misdiagnosed as pneumonia—manifested with pulmonary hemorrhage and was ultimately confirmed via CT-guided pleural biopsy, underscoring the limitations of fluid cytology. Early tissue diagnosis is essential to avoid treatment delays in this highly aggressive tumor. Further research into targeted and immune-based therapies is needed to improve the currently dismal prognosis.

## Patient consent

Written, informed consent was obtained from the patient for the publication of this case report. The patient has been informed that their case will be published in a medical journal for educational and scientific purposes. No identifiable personal information has been included in the manuscript to ensure patient privacy and confidentiality.
